# Non-immune Intravascular Hemolytic Anemia, an Unusual Presentation of Severe Vitamin B-12 Deficiency

**DOI:** 10.7759/cureus.26507

**Published:** 2022-07-02

**Authors:** ELMustafa Abdalla, Anas Al-Sadi, Abdalla Fadul, Ahmed H Ahmed, Muzamil Musa

**Affiliations:** 1 Internal Medicine, Hamad Medical Corporation, Doha, QAT

**Keywords:** anemia, cobalamin deficiency, rare clinical entity, vit b12 deficiency, non-immune hemolytic anemia

## Abstract

Vitamin B12 deficiency is a multifactorial condition, with a wide range of clinical presentations from mild to severe anemia and anemia-related neurological deficits. Hemolysis is a unique cause but has increasingly been recognized lately as a possible B12 deficiency presentation. Our patient presented with hemolytic anemia, for which extensive workup has excluded the common hemolysis etiologies. Therefore, it was attributed to B12 deficiency and improved significantly after treatment. Our case highlights the significance of this unusual presentation and its clinical implementation.

## Introduction

Vitamin B12 is essential for hematological, cardiovascular, and neurocognitive functions. Its deficiency can be caused by various factors, including malabsorption, immunological, or dietary deficiencies. Age, heredity, and lifestyle all substantially impact particular kinds of vitamin B12 deficiency [1]. Its specific structure and composition play a variety of roles at several cellular levels, including DNA and red blood cell (RBC) production, as well as in other neurologic processes [2]. Vitamin B12 deficiency symptoms range from tiredness, glossitis, and subtle neurologic impairment in mild-to-moderate cases to severe hematological problems, neurological manifestations, and cardiomyopathy in severe instances [3]. Concurrent hemolysis has been linked to intramedullary destruction of RBC (ineffective erythropoiesis) in individuals with Vitamin B12 deficiency [4]. Herein, we report a case of a young gentleman who presented with epigastric pain, a lack of vitamin B12, and hemolytic anemia.

## Case presentation

A 27-year-old Indian male patient, strictly vegan for religious reasons, presented with abdominal pain of one-day duration. The pain was mainly in the epigastric area, not radiated, and associated with vomiting of food content five times without blood or coffee-ground material. His friends noticed yellow discoloration in his eyes for 10 days. A review of systems was notable for feeling fatigued with mild exertional shortness of breath for several months. He denied any weight loss, change in bowel habits, fever, alcohol intake, recent herbal/medication use, or previous similar attacks. Family history was insignificant, and there was no recent travel, infection, bleeding tendency, or sick contact.

Examination showed scleral icterus and mucosal pallor. Vital signs were normal, and the abdomen was soft and lax with mild epigastric tenderness. No organomegaly was appreciated, and no abnormal lymph node was identified with normal heart and respiratory examination.

The initial investigations are shown in Table 1.

**Table 1:** The initial basic investigations INR: international normalized ratio; APTT: activated partial thromboplastin time; ALT: alanine transaminase; AST: aspartate aminotransferase

Haemoglobin	4.6 gm/dl (normal 12-15)
Haematocrit	13.5% (normal 40-50)
White blood cells	4700 /uL (normal 4-10)
Platelets	167000 /uL (normal 150000-400000)
Mean corpuscular volume (MCV)	110.4 fL (normal 80-100)
Mean corpuscular hemoglobin concentration (MCHC)	36.8 pg (normal 27-32)
RBC distribution width (RDW)	16.6 (normal 11-14)
Prothrombin time	11.8 seconds (normal 9/.7-11.8)
INR	1.1
APTT	23.6 seconds (normal 24.6-31.2)
Total protein	65 gm/L (normal 60-80)
Albumin level	39 gm/L (normal 35-50)
Alkaline phosphatase	51 U/L (normal 40-129)
ALT	19 U/L (normal 0-41)
AST	38 U/L (normal 0-40)

Once anemia was confirmed with the repeated sample, the patient was admitted as a case of severe anemia for evaluation. His total bilirubin was 87 umol/L (normal up to 21) with a direct bilirubin of 7 umol/L, lactate dehydrogenase (LDH) was 1500 U/L (normal up to 225), and haptoglobin was <10 mg/dl (normal range 30-200). international normalized ratio (INR) was 1.1, corrected reticulocyte count was 2.8%, calculated reticulocyte production index was 0.31%, and renal and other liver function parameters were normal (see Table 1). 

Because of these findings, hemolytic anemia was diagnosed, and a peripheral smear showed severe macrocytic anemia with anisopoikilocytosis, anisochromia, macroovalocytes, some tear drops, spherocytes, target cells, and fragmented red cells with some hypersegmented neutrophils.

A thorough evaluation was done to clarify the cause of this severe hemolytic anemia that was remarkable only for deficient vitamin B12 <73 pmol/L (normal 145-596). Direct and indirect Coomb’s test, G6PD, hemoglobin electrophoresis, iron profile, folate, and thyroid function test all were within the standard limit (Table 2).

**Table 2:** The results of further anemia work-up

Ferretin	170 ug/L (normal 38-270)
Iron	46 umol/L (normal 6-35)
Fe% Saturation	87% (normal 15-45)
Folate	16 nmol/L (normal 10-70)
G6PD	468 mU/109 RBC (normal 191-327)
Thyroid-stimulating hormone (TSH)	2.92 mIU/L (normal 0.3-4.2)
Coomb’s test	Negative
Prothrombin time	11.4 seconds (normal 9.7-11.8)
Hgb A	95.8 % (normal 95.8-98.0)
Hgb A2	3.2 % (normal 2.0-3.3)
Hgb F	0.6 % (normal 0.0-0.9)
Hgb S	0.0 %
Hgb H	Absent

Screening for pernicious anemia with anti-parietal cell and anti-intrinsic factor was unremarkable. Ultrasound abdomen ruled out hepatosplenomegaly and cholelithiasis. Abdominal pain subsided completely with simple analgesia and proton pump inhibitor, and he became asymptomatic within 24 hours of admission.

The patient received two units of packed red blood cells; he was also started on cyanocobalamin (Vitamin B12) 1000 mcg daily injection for one week, then shifted to oral cyanocobalamin. Upon discharge, he was asymptomatic, his hemoglobin improved to 6.9 gm/dl (from 4.6 gm/dl), and bilirubin dropped to 37 umol/L (from 87 umol/L).

Follow-up after three weeks showed complete resolution of anemia with hemoglobin of 13.6 gm/dL, with a mean corpuscular volume of 95.4 fL. The patient insisted on continuing his vegan diet and was instructed to keep on vitamin B12 supplements for life.

## Discussion

Anemia is defined as the decreased oxygen-carrying capacity of the blood. The underlying three pathophysiologic states that can contribute to anemia include (1) blood loss, (2) erythropoiesis failure, and (3) erythrocyte destruction. Anemia is characterized as microcytic, normocytic, or macrocytic based on the measured red cell size (MCV) [5].

During the diagnostic evaluation of both normocytic and macrocytic anemia, hemolytic anemia should be included in the differential diagnosis. Hemolysis, defined as premature red blood cell destruction, can happen intravascularly, in the reticuloendothelial system (extravascular), or both. Patients with hemolysis may usually present with acute anemia, jaundice, hematuria, dyspnea, tiredness, tachycardia, and potentially hypotension. Laboratory test results that support the presence of hemolysis are reticulocytosis, increased LDH, unconjugated bilirubin, and reduced haptoglobin levels. The direct antiglobulin test helps distinguish immunological from non-immune causes [6]. Our patient presented with exertional dyspnea, extreme fatigue, nausea, vomiting, and abdominal pain with co-existing extremely low hemoglobin of 4.6 mg/dl, jaundice, elevated indirect bilirubin, and lactate dehydrogenase with low haptoglobin, confirming hemolytic anemia as a cause.

Hemolysis can occur due to different causes ranging from medications to autoimmune and viral diseases to vitamin B12 or folate deficiencies. In up to 10% of those affected, a vitamin B12 deficiency can cause hemolysis [7]. Cobalamin is a significant element essential for the metabolic processes involved in synthesizing DNA and RNA as it is involved in tetrahydrofolate regeneration; thus, vitamin B12 deficiency will lead to ineffective hematopoiesis, causing hemolysis. This is an additional rationale for hemolysis, although unclear why vitamin B12 deficiency can lead to intramedullary destruction of RBC [8]. Moreover, vitamin B12 is essential in eradicating homocysteine by converting it to methyl methionine, which can lead to hemolysis if elevated [9].

Vitamin B12 is a water-soluble vitamin found in various foods, including dairy products and beef. The terminal ileum absorbs B12 after binding to intrinsic factors generated by stomach parietal cells during digestion. Vitamin B12 is essential for DNA synthesis, hematopoietic cell division, and appropriate neurologic function [10].

Inadequate intake, bioavailability, or malabsorption all contribute to B12 deficiency. Intracellular deficit of B12 occurs when B12 transport in the blood is disrupted, or cellular absorption or metabolism is impeded [11].

## Conclusions

This case report reveals the unusual but serious manifestations of vitamin B12 insufficiency. It reminds clinicians that when they see hemolysis for no obvious reason, vitamin B12 insufficiency should be investigated. It also emphasizes that vitamin B12 supplementation can help correct these outcomes.
